# Drainless Lipoabdominoplasty Using Progressive Tension Sutures, Scarpa's Fascia Preservation, and Minimal Cautery in 350 Consecutive Cases

**DOI:** 10.1093/asjof/ojag023

**Published:** 2026-03-13

**Authors:** R Brannon Claytor, Patricia M Fuentes, Grace C Tolan, Michael Karon

## Abstract

**Background:**

Lipoabdominoplasty has soared in popularity. Advancements in the procedure have led to improved abdominal contours. Despite improvements in surgical techniques, postoperative complications persist, especially seroma formation. Drains are commonly used to reduce the risk of seroma formation; however, drains are a nuisance for patients and may contribute to long-term complications. Many surgical strategies have been proposed to reduce seroma formation in lipoabdominoplasty, including progressive tension sutures (PTS), minimal cautery, and preservation of Scarpa's fascia; however, these strategies in combination remain underexplored.

**Objectives:**

The objective of this study was to determine the postoperative seroma rate of lipoabdominoplasty with combined PTS, minimal cautery, and Scarpa's fascia preservation without postoperative drains.

**Methods:**

This is a review of 350 patients who underwent drainless lipoabdominoplasty performed by a single surgeon from January 2017 to December 2024 at a single surgery center. Patient demographics, operative characteristics, and postoperative outcomes were collected. Descriptive analysis was performed. Pearson's χ^2^ and Mann–Whitney *U* tests were used to compare groups. Multiple logistic regression analysis was performed. Statistical significance was set at *P* < .05.

**Results:**

Among 350 cases, a total of 286 full abdominoplasties, 50 belt lipectomies, and 14 modified float abdominoplasties were performed. There was a total of 32 (9.1%) complications, with a 2% seroma rate. There were no mortalities. All patients who developed complications recovered without sequelae.

**Conclusions:**

The drainless lipoabdominoplasty can be performed safely with a low seroma rate using a multimodality approach that includes progressive tension sutures, minimal cautery, and preservation of Scarpa's fascia.

**Level of Evidence: 4 (Risk):**

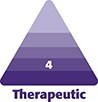

Abdominoplasty is one of the most commonly performed plastic surgery procedures, with 171,064 cases reported in 2024 alone.^1^ Increased demand for lipoabdominoplasty has resulted in a focus on refining methods to optimize patient aesthetic outcomes while minimizing the risk of postoperative complications, most commonly seroma.^2,3^ Although estimates vary, the accepted standard incidence of seroma is 10%.^4^ Seroma can lead to serious complications such as infection, wound dehiscence, flap necrosis, pseudocyst formation, and reoperation. Placement of postoperative drains has been the mainstay prophylaxis for seroma formation.^5,6^ Although effective at extravasating fluid accumulations, drains have been associated with impairment in quality of life, limitation in range of motion, increased wound infections, and decreased overall patient satisfaction.^7,8^

Surgical ingenuity and refinements aimed at achieving the ideal abdominal contour without the need for drains have progressed beyond traditional excision alone to excision combined with liposuction, progressive tension sutures (PTS), discontinuous undermining, preservation of Scarpa's fascia, and minimal electrocautery.^9-11^ The addition of liposuction has benefited patients by removing subcutaneous fat and sculpting a toned abdominal contour.^12-15^ Surgical methods such as preservation of Scarpa's fascia and discontinuous undermining have been adopted to safeguard the abdominal lymphatic structures, thereby decreasing fluid extravasation.^16,17^ Techniques like PTS, intended to reduce the shearing forces between the abdominal wall and flap tissue, have been shown to significantly decrease the frequency of seroma formation.^12,18-23^ Friedman et al found that minimal use of cautery lessens thermal injury to the abdominal region, therefore mitigating the activation of inflammatory pathways and fluid accumulation. However, the effect of these techniques in combination remains underexplored in the literature. Therefore, the objective of this study was to analyze the postoperative seroma rate in drainless lipoabdominoplasty performed using PTS, minimal cautery, and preservation of Scarpa's fascia in a single surgery center.

## METHODS

This was a retrospective study approved by the Main Line Health Institutional Review Board (E-24-5419). Patients were identified as individuals who underwent drainless full abdominoplasty, belt lipectomy, or modified float abdominoplasty, performed by the primary surgeon (RBC) between January 2017 and November 2024.

### Patient Selection

All patients included in the study underwent procedures involving plication of the rectus muscles with or without concomitant elective surgical procedures. Patients were excluded from the study if they had reverse abdominoplasty or mini abdominoplasty. There were no BMI or comorbidity restrictions. If patients were considered “high risk” (Caprini score > 6) for venous thromboembolism based on the 2005 Caprini Risk Assessment Model, a hematology consult was required before their operation per the primary surgeon's preferred venous thromboembolism (VTE) prophylaxis protocol. No patient that underwent hematology consultation was excluded from the study. Any patient on hormones was instructed to discontinue medication 2 weeks before surgery.

### Data Collection and Statistical Analysis

All demographic data, intraoperative metrics, and postoperative outcomes were collected. The procedure type was further subdivided into full abdominoplasty, belt lipectomy, and modified float abdominoplasty. The primary postoperative outcome collected was defined as any seroma that required aspiration. Secondary outcomes collected included hematoma, wound infection, wound-healing complications, and pulmonary embolism.

All analyses were performed using IBM SPSS Statistics version 30.0 (IBM Corporation, Armonk, NY). Descriptive statistics were used to summarize patient demographics, intraoperative metrics, and postoperative outcomes. Categorical variables were evaluated through Pearson's χ^2^ tests, and continuous variables were assessed through the Mann–Whitney *U* test. Multiple logistic regression analysis was conducted to account for potential cofounders. Statistical significance was set at *P* < .05.

### Surgical Technique

#### Preoperative Guidelines

Preoperatively, all patients received a 5000-unit Heparin injection and intermittent pneumatic compression (IPC) devices which were also worn throughout the entire procedure, following the primary surgeon's VTE chemoprophylaxis protocol.^24^ Low/moderate-risk patients (Caprini 2-5) received enoxaparin injections for 7 days postoperatively unless the primary surgeon determined there were signs of ecchymosis in which case the enoxaparin was held for 24 h. High-risk patients (Caprini >6) continued enoxaparin injections for 30 days after surgery.

#### Full Abdominoplasty

The present surgical technique was performed by the primary surgeon, a board-certified plastic surgeon, with 17 years of experience practicing drainless lipoabdominoplasty.

Patients were marked in an upright position. Patients were placed under general anesthesia. Dependent on patient BMI, a range of 3 to 8 L solution of 1000 mL normal saline, 1 mg of epinephrine (1:1000), and 20 mL of 1% lidocaine (200 mg) was infiltrated under the abdominal skin. Using 3, 4, and 5 mm cannulas, the areas to be suctioned were pretunneled and subsequently suctioned in a fan-like fashion using power-assisted liposuction. The infraumbilical flap is liposuctioned to accelerate flap removal, and the upper flap is liposuctioned to preserve lymphatic channels and contour aesthetic results.

The primary surgeon's technique for full lipoabdominoplasty includes en bloc excision of marked infraumbilical adipocutaneous flap and preservation of Scarpa's fascia laterally and centrally, while excising to the level of the rectus fascia from the infraumbilical flap to the flap at the level of the xyphoid.

All tissue excised and dissected is done through sharp dissection with minimal use of cautery. Then, elevation of the abdominal flap to the level of the xiphoid is performed by sharp dissection with #10 blade scalpel with minimal use of electrocautery for hemostasis. Identification of rectus diastasis at the medial border of the rectus muscle is marked. Plication of the rectus muscles is performed with interrupted figure of eight 0 Prolene (Ethicon Inc., Cornelia, GA) sutures, running 2-0 Prolene, and a final layer of running 2-0 PDS from the xiphoid to the umbilicus and from the umbilicus to the mons pubis.

Finally, superficial PTS with 2-0 Vicryl (Ethicon Inc., Cornelia, GA) are placed from the midline of the upper flap to the suture line of the rectus plication repair down to the level of the umbilicus. Multiple interrupted 2-0 Vicryl sutures are placed throughout the abdomen to advance the lateral flap more medially for abdominal contour as well as to close off any open space inferior to the umbilicus. See Video for the full abdominoplasty surgical technique.

**Figure 1.**
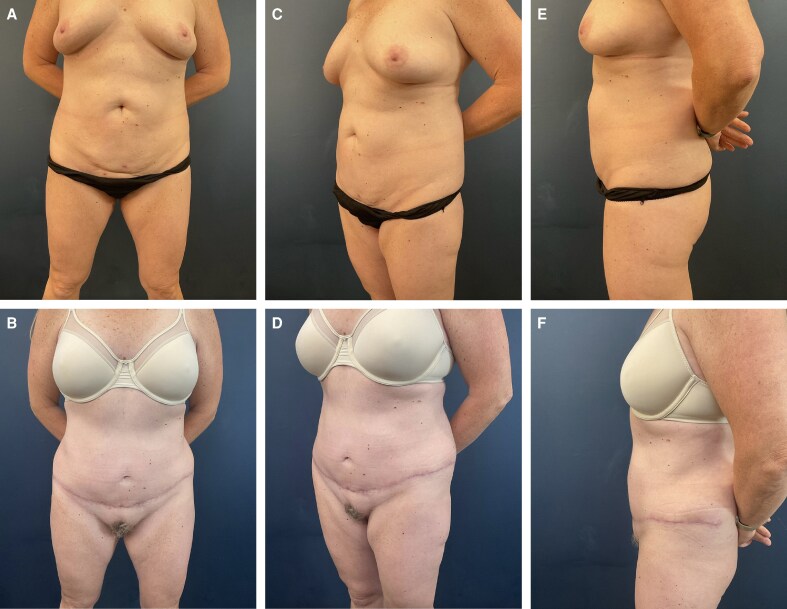
Patient is a 58-year-old female 6 months after modified float lipoabdominoplasty. (A) Preoperative anterior view. (B) Postoperative anterior view. (C) Preoperative anterior oblique view. (D) Postoperative anterior oblique view. (E) Preoperative lateral view. (F) Postoperative lateral view.

**Figure 2.**
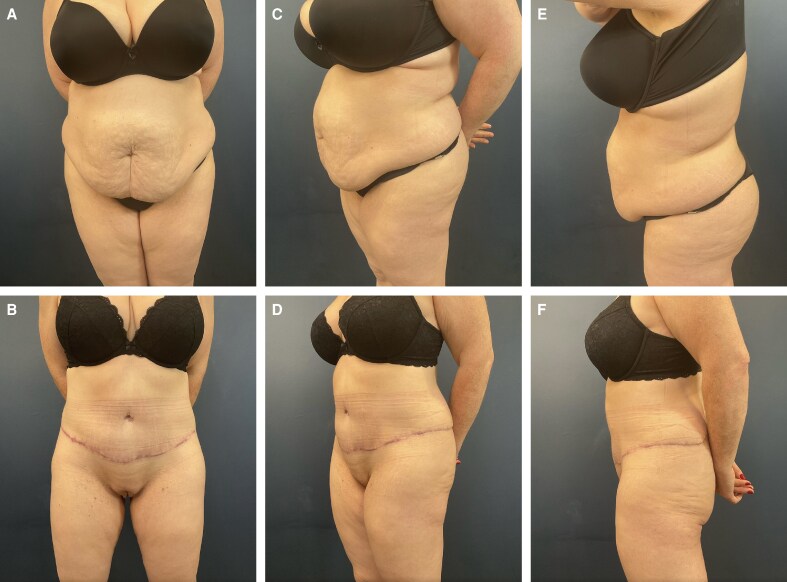
Patient is a 43-year-old female 8 months after full lipoabdominoplasty. (A) Preoperative anterior view. (B) Postoperative anterior view. (C) Preoperative anterior oblique view. (D) Postoperative anterior oblique view. (E) Preoperative lateral view. (F) Postoperative lateral view.

**Figure 3.**
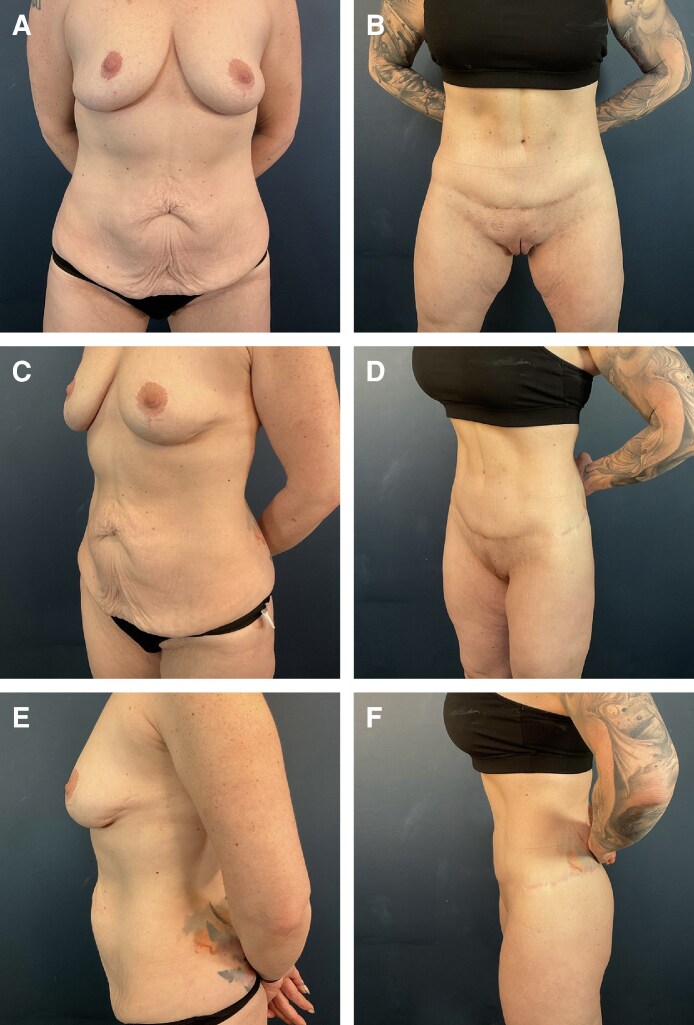
Patient is a 45-year-old female 2 years after belt lipectomy. (A) Preoperative anterior view. (B) Postoperative anterior view. (C) Preoperative anterior oblique view. (D) Postoperative anterior oblique view. (E) Preoperative lateral view. (F) Postoperative lateral view.

**Figure 4.**
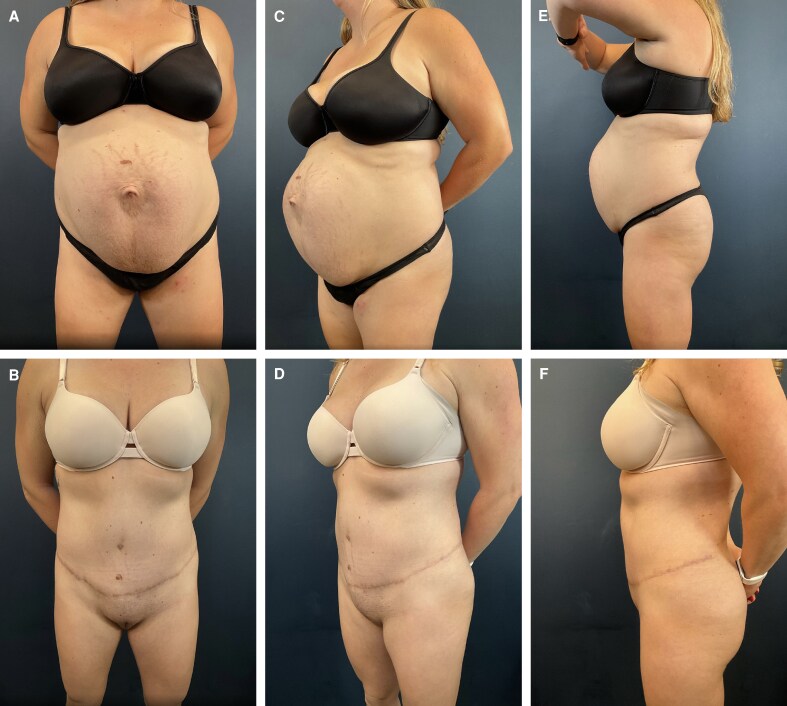
Patient is a 37-year-old female 9 months after full lipoabdominoplasty. (A) Preoperative anterior view. (B) Postoperative anterior view. (C) Preoperative anterior oblique view. (D) Postoperative anterior oblique view. (E) Preoperative lateral view. (F) Postoperative lateral view.

To address loose thigh skin, medial and lateral Scarpa's fascia is grasped and advanced superiorly and inset into fascia to decrease tension of the wound closure and address existing loose skin and cellulite of the thighs. Deep closure is performed with a 3-0 PDS followed by dermal closure with 3-0 barbed Monocryl and the subcuticular layer is closed with 4-0 Monocryl. Wounds are dressed with Dermabond glue. No drains are placed. Preoperative and postoperative results can be seen in Figures 1-4.

#### Modified Float Abdominoplasty

Similar to the full abdominoplasty, with the exception that the umbilicus is maintained at the level of skin. The umbilicus stalk is released from the abdominal fascia. Any hernia created is repaired with 2-0 Prolene. All other surgical steps are similar to full abdominoplasty. The umbilicus is re-inset into the rectus fascia with 3-0 Prolene. No drains are placed.

#### Belt Lipectomy

For a belt lipectomy, the patient is first placed prone, and the posterior section is excised to the level of Scarpa's fascia. No undermining of the buttock flap is performed. All wounds are closed with interrupted 0-PDS in deep layers to completely eliminate any open space and 3-0 PDS at deep dermis with 3-0 Monocryl subcutaneously. The patient is then rotated into supine position and completion of abdominoplasty is performed. No drains are placed.

#### Postoperative Care

All patients are placed in an abdominal binder, which is fitted and marked before the patient is transferred to recovery. Patients were provided with IPC devices before discharge and were instructed to wear them for at least 2 weeks postoperatively. This is a low cost and low risk postoperative intervention in the primary surgeon's VTE prophylaxis plan. All patients were discharged on the same day as their procedure. At 24 h postoperatively, patients are instructed to upload images of their incisions to TouchMD (TouchMD, Cedar City, UT) for evaluation by the primary surgeon.

If there is minimal ecchymosis, the patient is instructed to start 1 week of 40 mg enoxaparin injections. If the primary surgeon determines there is extensive ecchymosis, enoxaparin is held for an additional 24 h to reduce potential for hematoma formation or other bleeding complications. Minimal ecchymosis was defined as bruising local to the incision site, whereas extensive ecchymosis was characterized as bruising extending from the incision site down to the knee and/or up to the breast. If the patient is considered high risk by the 2005 Caprini risk assessment model (score > 6), patients continue enoxaparin injections for 30 days postoperatively, per the primary surgeon's previously published chemoprophylaxis algorithm for VTE in lipoabdominoplasty.^24^ Patients are evaluated in the clinic weekly for the first month postoperatively, then monthly for 6 months, and then biannually.

## RESULTS

This study includes 350 patients from January 2017 to December 2024 who underwent drainless lipoabdominoplasty which included full abdominoplasty, belt lipectomy, and modified float abdominoplasty. The study group had an average age of 45.82 years (range, 24-78), with an average BMI of 27.54 kg/m^2^ (range, 18.2-43.3). The average operative time was 258.56 min (range, 120-485 min). Forty-four (12.6%) patients reported current use of oral or systemic hormones and were instructed to stop usage 2 weeks before surgery. All demographic and operative characteristics are included in Tables 1-4.

**Table 1.** Demographic and Operative Characteristics of Study Group (*n* = 350)

Demographic and operative characteristics (full abdominoplasty, belt lipectomy, float modification)	
Age (years)	
Mean ± SD	45.82 ± 10.0
Range	24-78
Gender	Female: 336
	Male: 14
BMI (kg/m^2^)	
Mean ± SD	27.54 ± 4.8
Range	18.2-43.3
Caprini score	
Mean ± SD	3.9 ± 1.2
Range	2-9
Current smoker (yes)	0
Diabetes mellitus (Type 1 or Type 2)	5 (1.4%)
Hormone use (yes)	44 (12.6%)
Preoperative hematology consult	28 (8%)
Operative time (min)	
Mean ± SD	258.56 ± 69.5
Range	120-485
Liposuction amount (mL)	
Mean ± SD	1122.56 ± 714.0
Range	20-4000
Weight of tissue (g)	
Mean ± SD	2,102.92 ± 1,365.4
Range	130-12,700

SD, standard deviation.

**Table 2.** Demographic and Operative Characteristics of Patients Who Underwent Full Abdominoplasty (*n* = 286)

Demographic and operative characteristics (full abdominoplasty)	
Age (years)	
Mean ± SD	45.7 ± 10.0
Range	24-78
BMI	
Mean ± SD	27.4 ± 4.7
Range	18.2-43.3
Caprini score	
Average	3.8
Range	2-8
Operative time (min)	
Mean ± SD	245.7 ± 60.3
Range	120-459
Liposuction amount (mL)	
Mean ± SD	1,052.1 ± 652.5
Range	20-4,000
Weight of tissue (g)	
Mean ± SD	1,950.3 ± 1,085.2
Range	150-5,443

SD, standard deviation.

**Table 3.** Demographic and Operative Characteristics of Patients Who Underwent Belt Lipectomy (*n* = 50)

Demographic and operative characteristics (belt lipectomy)	
Age (years)	
Mean ± SD	46.0 ± 10.1
Range	30-64
BMI (kg/m^2^)	
Mean ± SD	29.8 ± 4.4
Range	22-40.4
Caprini score	
Average	4.2
Range	3-9
Procedure length (min)	
Mean ± SD	399.9 ± 65.4
Range	141-485
Liposuction amount (mL)	
Mean ± SD	1,629.8 ± 846.5
Range	300-4,000
Weight of tissue (g)	
Mean ± SD	3,335.4 ± 2,020.2
Range	250-12,700

SD, standard deviation.

**Table 4.** Demographic and Operative Characteristics of Patients Who Underwent Modified Float Abdominoplasty (*n* = 14)

Demographic and operative characteristics (modified float abdominoplasty)	
Age (years)	
Mean ± SD	45.8 ± 9.7
Range	33-61
BMI (kg/m^2^)	
Mean ± SD	22.9 ± 3.9
Range	19.3-32.1
Caprini score	
Median	3
Range	2-4
Procedure length (min)	
Mean ± SD	236.3 ± 73.7
Range	120-349
Liposuction amount (mL)	
Mean ± SD	702 ± 698.5
Range	100-2000
Weight of tissue (g)	
Mean ± SD	375 ± 284.7
Range	130-800

SD, standard deviation.

### Postoperative Complications

There were a total of 32 (9.1%) complications in the study group (Table 5). Seroma occurred in 2% (*n* = 7) of patients who were treated with in-office aspiration without any cases of reoperation. Distribution by procedure type included 6 cases of seroma in the full abdominoplasty group and 1 case in the modified float abdominoplasty group. The weight of tissue excised was associated with seroma formation (*P* = .022*; Table 6). Secondary complications in the study group included wound-healing complications that occurred in 2.6% (*n* = 9), wound infection in 2.3% (*n* = 8), hematoma in 1.7% (*n* = 6), and pulmonary embolism in 0.57% (*n* = 2). No revision surgeries were performed. All patients recovered with no long-term sequelae.

**Table 5.** Postoperative Complications by Procedure Type

	Full abdominoplasty (*n* = 286)	Belt lipectomy (*n* = 50)	Modified float (*n* = 14)	Total (*N* = 350)
Overall complications	27 (9.4%)	4 (8.0%)	1 (7.1%)	32 (9.1%)
Seroma	6 (2.1%)	0 (0.0)	1 (7.1%)	7 (2.0%)
Wound healing	8 (2.8%)	1 (2.0%)	0 (0.0)	9 (2.6%)
Wound infection	6 (2.1%)	2 (4.0%)	0 (0.0)	8 (2.3%)
Hematoma	5 (1.7%)	1 (2.0%)	0 (0.0)	6 (1.7%)
Pulmonary embolism	2 (0.7%)	0 (0.0)	0 (0.0)	2 (0.6%)

**Table 6.** Univariate Analysis of Non-Seroma and Seroma Groups

Variable (Mean ± SD)	Nonseroma group	Seroma group	*P*-value
Age (years)	46.80 ± 9.97	46.71 ± 11.16	.891
BMI (kg/m^2^)	27.48 ± 4.72	30.31 ± 7.15	.216
Operative time (min)	258.97 ± 69.69	238.29 ± 62.52	.462
Liposuction volume (mL)	1117.33 ± 708.60	1440 ± 1045.47	.531
Weight of tissue resected (g)	2084.48 ± 1366.92	3180 ± 749.67	.022*

SD, standard deviation. *Statistical significance set at *P* < .05.

A multivariable logistic regression analysis was performed to identify factors associated with the occurrence of postoperative complications across all procedure types. The model included patient age, BMI, operative time, primary abdominoplasty technique, presence of additional procedures, Caprini score, liposuction volume, and tissue weight resected. An increase in the weight of tissue excision was associated with an increased risk of postoperative complications (aOR 1.000, *P* = .044*; Table 7).

**Table 7.** Weight of Tissue Resected is Associated With an Increase in Postoperative Complications in Multiple Logistic Regression

Variable	aOR (95% CI)	*P*-value
Age (years)	1.008 (0.965-1.052)	.725
BMI (kg/m^2^)	1.036 (0.935-1.149)	.495
Operative time (min)	0.999 (0.992-1.007)	.828
Additional procedures	0.830 (0.344-2.001)	.678
Caprini score	0.853 (0.587-1.239)	.405
Liposuction amount (mL)	1.000 (0.999-1.001)	.703
Weight of tissue (g)	1.000 (1.000-1.001)	.044*

aOR, adjusted odds ratio. *Statistical significance set at *P* < .05.

### Planned Combined Procedures

There were 186 (53.1%) patients who underwent additional procedures, and bilateral mastopexy was the most commonly performed at 31.18% (*n* = 58; Table 8). The complication rate in patients who underwent additional procedures was 10.4% (*n* = 23). Undergoing an additional procedure with lipoabdominoplasty was not associated with an increase in postoperative complications (*P* = .678).

**Table 8.** Additional Procedures Performed at the Time of the Primary Procedure

Total additional procedures	186 (53.1%)
Bilateral mastopexy	58 (31.2%)
Umbilical hernia repair	49 (26.3%)
Liposuction of upper flank	35 (18.8%)
Liposuction of trunk	33 (17.7%)
Breast augmentation	32 (17.2%)
Neo-umbilicus	21 (11.3%)
Fat graft to breast	13 (6.9%)
Liposuction of the thighs	12 (6.5%)
Ventral hernia repair	11 (5.9%)
Fat graft to buttock	10 (5.4%)
Breast reduction	8 (4.3%)
Breast implant replacement	5 (2.7%)
Liposuction of chest	4 (2.1%)
Liposuction of arms	4 (2.1%)
Gynecomastia	3 (1.6%)
Fat graft to face	2 (1.1%)
Facelift	2 (1.1%)
Brachioplasty	2 (1.1%)
Blepharoplasty	2 (1.1%)
Liposuction of neck	2 (1.1%)
Breast implant removal	1 (0.5%)
Lumpectomy	1 (0.5%)
Lateral trunkplasty	1 (0.5%)
Skin lesion excision	1 (0.5%)
Necklift	1 (0.5%)

The values are expressed as *n* (%).

## DISCUSSION

Lipoabdominoplasty is indicated for patients with excess abdominal skin laxity, lipodystrophy, rectus abdominis diastasis, and for contouring after massive weight loss.^25^ Owing to its ability to achieve significant abdominal reshaping and simultaneously address skin redundancy and excess adipose, its popularity has continued to rise.^26^ As demand grows, minimizing postoperative complications becomes increasingly important. This single-center study evaluated postoperative seroma rates following drainless lipoabdominoplasty performed with intraoperative progressive tension sutures (PTS), minimal thermal injury, and preservation of Scarpa's fascia. We observed a low overall seroma rate, suggesting that lipoabdominoplasty can be performed safely without postoperative drains when these operative modifications are employed in combination.

Seroma remains the most frequent complication after abdominoplasty, with reported rates up to 10%.^4,23^ Drains are the mainstay for seroma prevention but carry disadvantages, including discomfort, mobility restriction, and increased risk of infection.^5,6,10,27^ Despite these limitations, drains are widely used, with 82.5% of plastic surgeons indicating frequently using drains in their practice, although this marks a decline from 98% in 2006 in a national study.^28^ Although drains help manage fluid buildup, patient dissatisfaction with their use continues to be a concern. The low seroma rate in this study corroborates the notion that alternative strategies to manage seroma formation may offer improved patient comfort without compromising safety. Moreover, this was observed across all approaches included in this study.

Progressive tension sutures, first introduced by Pollock and Pollock in 2000, have demonstrated significant efficacy in reducing seroma formation by eliminating dead space and distributing tension between the abdominoplasty flap and musculoaponeurotic abdominal wall.^18,29^ This technique has gained substantial support in the literature.^30^ A systematic review by Rao et al concluded that PTS were associated with lower seroma rates compared with the use of drains alone.^31^ Similarly, Li and Wang conducted a meta-analysis and found that patients in the PTS-only group had a significantly lower incidence of seroma than those managed with drains alone.^32^ In the present study, PTS were placed from the xiphoid to the pubic region to minimize dead space. This approach is further supported by findings from Bromley et al, who reported that patients who developed seromas without receiving PTS required more frequent aspirations. Their data suggests not only a higher incidence of seroma in the absence of PTS but also a greater volume and recurrence of fluid accumulation when PTS are omitted.^33^ Collectively, these findings reinforce the role of PTS as an effective strategy for seroma prevention, eliminating the need for surgical drains. In addition to these benefits, PTS provides the surgeon with additional body sculpting and shaping techniques for improved definition of flanks and rectus muscle contours.

Scarpa's fascia preservation was also employed by the primary surgeon. The importance of preserving this fascial layer was highlighted by Friedman et al, who used immunohistochemical analysis to demonstrate that nearly 10% of abdominal lymphatics course through Scarpa's fascia. Thus, preserving this layer supports lymphatic function and may reduce fluid accumulation.^23^ Costa-Ferreira et al conducted a prospective cohort study of 160 patients undergoing abdominoplasty with and without Scarpa's fascia preservation. They found that patients in the preservation group had a statistically significant decrease in drain output, resulting in earlier drain removal.^34^ This supports the notion that preserving Scarpa's fascia helps maintain the integrity of local lymphatics and vessels, thereby promoting effective fluid drainage and respecting the physiology of the abdominal wall. Further supporting this, Xia et al also reported a significant reduction in seroma formation with Scarpa's fascia preservation. In their study, Scarpa's fascia was preserved both centrally and laterally during flap dissection.^35^ These findings underscore the role of Scarpa's fascia preservation in minimizing seroma risk.

In the present study, an increase in the weight of tissue resected was associated with an increased risk of seroma formation. This finding aligns with the current literature. Brown et al found in a retrospective cohort that patients who underwent high-resection weight cases were at an increased risk of seroma formation.^36^ Similarly, in a cohort of postbariatric patients, resection weight was identified as a predictor of postoperative complications.^37^ This relationship is likely multifactorial as larger resections create greater tissue disruption, increased lymphatic injury, and more potential dead space, all of which facilitate fluid accumulation. Despite this association, previous studies have demonstrated that PTS and Scarpa's fascia preservation can mitigate seroma risk even in high-resection weight cases.^38,39^ By securing the flap to the underlying facia, eliminating dead space, and maintaining the integrity of the abdominal lymphatic channels, these techniques may offset the challenges posed by high-weight resections. Our study further supports this concept, because overall seroma rates remained low.

Minimizing thermal injury is a subtle yet meaningful surgical refinement implemented by the primary surgeon. Although electrocautery is commonly used for hemostasis, it can induce localized thermal damage that provokes inflammation and promotes fluid exudation.^3,40^ To avoid these effects, the primary surgeon favors sharp dissection—a technique supported by evidence as having lower complication rates.^41^ The use of epinephrine-infused tumescent solution further enhances hemostasis, reducing the reliance on cautery. In a single-center prospective study, Valença-Filipe et al demonstrated a lower incidence of seroma with scalpel dissection compared with mono- or bipolar cautery in patients undergoing abdominoplasty. Similarly, Janis et al's systematic review found sharp dissection to be associated with the lowest seroma rates when compared with ultrasonic or electrocautery techniques. This is likely attributable, in part, to the preservation of the intricate lymphatic networks of the abdominal wall, which closely parallel the regional vasculature.^42^ Disruption of these vessels through thermal energy may impede lymphatic flow, increasing the risk of postoperative fluid accumulation. Although the present study does not isolate the specific impact of sharp dissection, the existing literature supports the concept that thermal injury triggers a proinflammatory cascade. Thermal injury to lymphatic vessels may impede lymphatic flow resulting in fluid accumulation thus raising interstitial pressure. This may result in compression of capillary networks and compromise tissue perfusion. The resulting lymphatic dysfunction contributes to greater fluid retention and elevated seroma risk.^41-43^

In the present study, most patients underwent at least 1 additional procedure, most commonly mastopexy, and found no association between postoperative complications and performing an additional procedure. The literature, however, has suggested that undergoing additional procedures with abdominoplasty increases the risk of postoperative complications.^44,45^ Winocour et al reported a 4.3% complication rate when abdominoplasty was performed alone and up to 10.4% with multiple combined surgeries. The results in the present study suggest that with appropriate patient selection, planning, and surgical execution, abdominoplasty combined with other procedures can be performed safely without increasing the risk of complications, which is especially important as these cases often involve longer operative times and extended recovery periods.

VTE, although rare, is a serious complication in abdominoplasty.^46^ The primary surgeon uses a tailored prophylaxis regimen, which indicates that all patients receive preoperative heparin and postoperative enoxaparin.^24^ Two patients (0.57%) developed postoperative pulmonary embolism, both of whom had a Caprini score of 4 and no known personal or family history of VTE. One patient presented to the emergency department 5 days after surgery following a syncopal episode, whereas the second presented with shortness of breath 28 days postoperatively. In both cases, CT imaging confirmed the diagnosis of pulmonary embolism. Both patients received appropriate treatment and recovered without sequelae. These findings are consistent with data in Keyes et al,^47^ reporting that many VTE events occur in patients with intermediate Caprini scores (2-8), illustrating the limitations of Caprini scoring alone. The PE incidence in this study is at the low end of previously reported rates (0.5%-2.3%).^48^ However, both events occurred in patients not typically considered high risk, highlighting the need for individualized assessment beyond standard scoring, with universal chemoprophylaxis and mechanical prophylaxis regardless of score.

Despite the strengths of this study, there are limitations worth considering. This study was conducted at a single surgical center and represents the experience of one surgeon, which limits generalizability. The small sample size limits the statistical power of the study, restricting the study's ability to draw definitive conclusions. Additionally, this study does not include a comparison group, thus definitive conclusions about the efficacy of a drainless approach cannot be drawn from the outcomes of this paper. Future studies should prioritize a multicenter or prospective cohort approach to validate the safety and effectiveness of drainless lipoabdominoplasty compared with a control group.

## CONCLUSIONS

Drainless lipoabdominoplasty is a highly sought out procedure; therefore, reducing postoperative complications is a priority. In this study, progressive tension sutures, meticulous sharp dissection preserving Scarpa's fascia, minimal cautery, and zero drains are combined in lipoabdominoplasty, resulting in a low seroma rate. This is a safe technique for drainless lipoabdominoplasty while maintaining patient comfort, satisfaction, and safety at the forefront.
